# Membrane Binding of MinE Allows for a Comprehensive Description of Min-Protein Pattern Formation

**DOI:** 10.1371/journal.pcbi.1003347

**Published:** 2013-12-05

**Authors:** Mike Bonny, Elisabeth Fischer-Friedrich, Martin Loose, Petra Schwille, Karsten Kruse

**Affiliations:** 1 Theoretische Physik, Universität des Saarlandes, Saarbrücken, Germany; 2 Max-Planck-Institut für Zellbiologie und Genetik, Dresden, Germany; 3 Max-Planck-Institut für Physik komplexer Systeme, Dresden, Germany; 4 Department of Systems Biology, Harvard Medical School, Boston, Massachussetts, United States of America; 5 Max-Planck-Institut für Biochemie, Martinsried, Germany; University of Connecticut, United States of America

## Abstract

The rod-shaped bacterium *Escherichia coli* selects the cell center as site of division with the help of the proteins MinC, MinD, and MinE. This protein system collectively oscillates between the two cell poles by alternately binding to the membrane in one of the two cell halves. This dynamic behavior, which emerges from the interaction of the ATPase MinD and its activator MinE on the cell membrane, has become a paradigm for protein self-organization. Recently, it has been found that not only the binding of MinD to the membrane, but also interactions of MinE with the membrane contribute to Min-protein self-organization. Here, we show that by accounting for this finding in a computational model, we can comprehensively describe all observed Min-protein patterns *in vivo* and *in vitro*. Furthermore, by varying the system's geometry, our computations predict patterns that have not yet been reported. We confirm these predictions experimentally.

## Author Summary

Cellular protein structures have long been suggested to form by protein self-organization. A particularly clear example is provided by the proteins MinC, MinD, and MinE selecting the center as site of cell division in the rod-shaped bacterium *Escherichia coli*. Based on binding of MinD to the cytoplasmic membrane and an antagonistic action of MinE, which induces the release of MinD into the cytoplasm, these proteins oscillate from pole to pole, where they inhibit cell division. Supporting the idea of self-organization being the cause of the Min oscillations, purified Min proteins were found to spontaneously form traveling waves on supported lipid bilayers. A comprehensive understanding of the Min patterns formed under various conditions remains elusive. We have performed a computational analysis of Min-protein dynamics taking into account the recently discovered persistent action of MinE. We show that this property allows to reproduce all observed Min-protein patterns in a unified framework. Furthermore, our analysis predicts new structures, which we observed experimentally. Our study highlights that mechanisms underlying the spontaneous formation of protein patterns under purified *in vitro* conditions can also generate patterns inside complex intracellular environments.

## Introduction

Nature presents an overwhelming variety of forms and patterns. While system specific conditions can play an important role for their formation, also a few general principles underlying biological pattern formation have been proposed in the past. A particularly attractive concept is the spontaneous formation of patterns in reaction diffusion systems as proposed by Alan Turing [1]. In this case, a (small) number of different constituents collectively form large-scale patterns. So far, however, only a few biological examples of bona fide Turing patterns are known [2].

**Figure 1:**
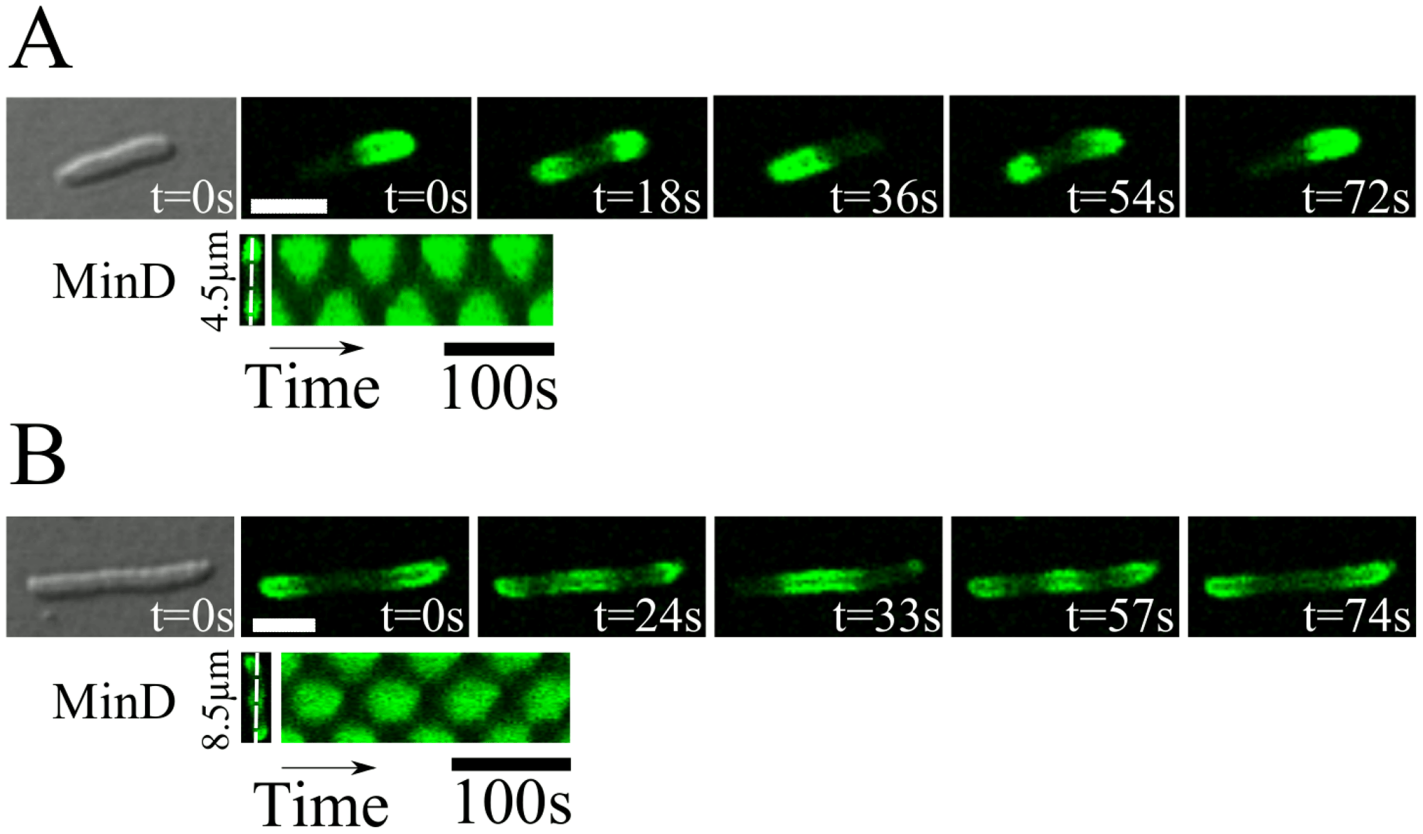
Different patterns formed by MinD in living *E. coli*. A) Standing wave with one node; B) standing wave with two nodes. Top: DIC image followed by snapshots from a time lapse recording of MinD-GFP; bottom: corresponding kymograph. Scale bar: 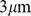.

An example of subcellular pattern formation due to reactions and diffusion of just two different constituents is provided by the Min system in the rod-shaped bacterium *Escherichia coli* [3]. This protein system forms a spatiotemporal oscillation in the cell, that is, a standing wave with a node in the cell center [4], [5], see Figure 1A, which plays an essential role in division site selection in *E. coli*. Whereas the oscillations emerge solely from the interactions between MinD, MinE and the membrane, the inhibitor of cell division MinC binds to MinD and is thus distributed similarly: it appears periodically at the cell poles, but is practically absent from the cell center. In this way, division occurs in the cell center leading to two daughter cells of the same size.

While some models suggest that particular properties of the cell poles might play an essential role for Min-protein pattern formation [6], a number of observations support the notion that the Min system can self-organize without any additional spatial cues. First of all, depending on the cell geometry and the Min-protein expression level, the protein pattern can change: In longer cells, standing waves with several nodes form [4], see Figure 1B, whereas in shorter cells and for slightly over-expressed Min proteins, oscillations are replaced by stochastic switching of the proteins between the two cell halves [7], [8]. In Y-shaped cells, the proteins visit the different arms in a way that depends on the arms' lengths [9].

Furthermore, in vitro studies of purified proteins found MinD and MinE to spontaneously organize into collective traveling waves [10]. Together, these observations suggest that the Min-protein patterns emerge from the intrinsic dynamics of these proteins, in particular, the exchange of proteins between the membrane, driven by the high affinity of MinD for the membrane when ATP is bound and a low affinity with ADP bound [11]. In addition, membrane-bound MinD recruits MinE, which in turn induces hydrolysis of the bound nucleotide by MinD and consequently MinD detachment from the membrane. These well-established processes are at the core of a number of computational models reproducing the Min-protein oscillations observed in E. coli [12].

The most popular mechanism studied through such models assumes that cooperative membrane-attachment of MinD is at the origin of pattern formation. In the simplest version, the rate of MinD attachment to the membrane increases in presence of membrane-bound MinD [13]. Several works on models implementing cooperative membrane attachment in various ways and complementing it by different side processes have shown that it can robustly generate the pole-to-pole oscillations observed in *E. coli* [14]–[16] even during septum closure [17]. Other works rather emphasized cooperative effects between already membrane-bound MinD [18], [19]. However, in spite of more than a decade of theoretical analysis, there exists to date no comprehensive description of all Min-protein patterns observed *in vivo* and *in vitro*.

Some evidence suggested that an N-terminal helix allows MinE to also interact with the membrane [20], [21], however, it remained unclear if this property was important for the self-organization of the Min system. Single molecule data obtained *in vitro* [22] as well as genetic, physiological, and structural analysis [23] finally provided evidence that the ability of MinE to interact with phospholipids allows it to remain bound to the membrane after MinD has detached, which could lead to the subsequent removal of several MinD dimers by one MinE dimer. In analogy to molecular motors that can perform several subsequent steps on a cytoskeletal filament, we call this property “MinE processivity”. This possibility had been proposed earlier on theoretical grounds as it offers a mechanism for the formation of MinE-rings [19], [24], [25] and was crucial for describing the guidance of Min-protein waves on patterned substrates [26]. In the present work, we perform a computational study to explore the consequences of this molecular property for large-scale pattern formation. To this end, we use deterministic and stochastic calculations in three dimensions. We show that MinE processivity provides a key to obtain a unified description of all previously described Min-protein patterns *in vivo* and *in vitro*. In addition, our analysis predicts hitherto unknown patterns, namely traveling waves in long and moving patches in aberrantly large cells. We confirm the existence of these states by fluorescence microscopy of living *E. coli* cells. Beyond the Min system, our findings highlight the importance of membrane-binding for subcellular pattern formation.

## Results

### Min-protein dynamics

#### Molecular interactions

We start by detailing the molecular interactions that we consider essential for understanding Min-protein pattern formation *in vivo* and *in vitro*, see also [27]. Let us start with the ATPase MinD. After binding ATP and in the presence of a lipid bilayer, an amphipathic helix is formed at the C-terminus of cytoplasmic MinD giving the protein an increased affinity for binding lipid bilayers [28]–[32]. Furthermore, ATP-binding leads to MinD dimerization. Only as a dimer, MinD has a sufficiently high affinity for binding to the cytoplasmic membrane. The binding kinetics of MinD shows deviations from Langmuir kinetics suggesting that MinD binding to the membrane is cooperative [30], [33], [34]. The molecular mechanism underlying cooperative MinD binding, though, is poorly understood.

Let us note that membrane-bound MinD can interact to form higher-ordered structures, however their exact lifetime and architecture is not known [35]–[37]. Experiments *in vitro* on vesicles incubated in a buffer containing MinD suggest a two-step process of MinD binding first to the membrane and subsequently forming clusters [35]. MinD proteins have been reported to arrange in a helical way [37]. It is not clear, though, whether aggregates of membrane-bound MinD play a functional role in Min-pattern formation. Note also, that recent works have provided evidence that the formation of MreB helices or foci of Clp Protease in *E. coli* were induced by attached fluorescent tags [38], [39]. It remains to be seen if a similar effect is responsible for the formation of MinD helices.

MinE and MinC are recruited to the cytoplasmic membrane by membrane-bound MinD dimers. They bind to overlapping sites located at the MinD-dimer interface [32], [40], [41]. At the same time MinE interacts directly with the membrane through an amphipathic *α*-helix [23]. The binding of MinE stimulates the ATPase activity of MinD and thus triggers the detachment of MinD from the membrane [29], [30]. Through its direct interaction with the membrane, MinE can reside on the membrane for a short period during which it can associate with another membrane-bound MinD dimer [22], [23]. Due to the interaction of a amphipathic N-terminal helix with the membrane, MinE is able to remain attached after activation and displacement of MinD to activate another MinD dimer bound to the membrane. Since the formation of this helix of MinE depends on the formation of a complex with its substrate MinD, this behavior is comparable to processive enzymes, which are able to remain attached to their substrates and perform a large number of rounds of catalysis before dissociating [42].

#### Molecular processes and dynamic equations

From the molecular interactions sketched above, we inferred the dominant reaction paths governing the macroscopic dynamics of the Min-protein distributions. To keep our description simple, we only considered MinD dimers.

**Figure 2:**
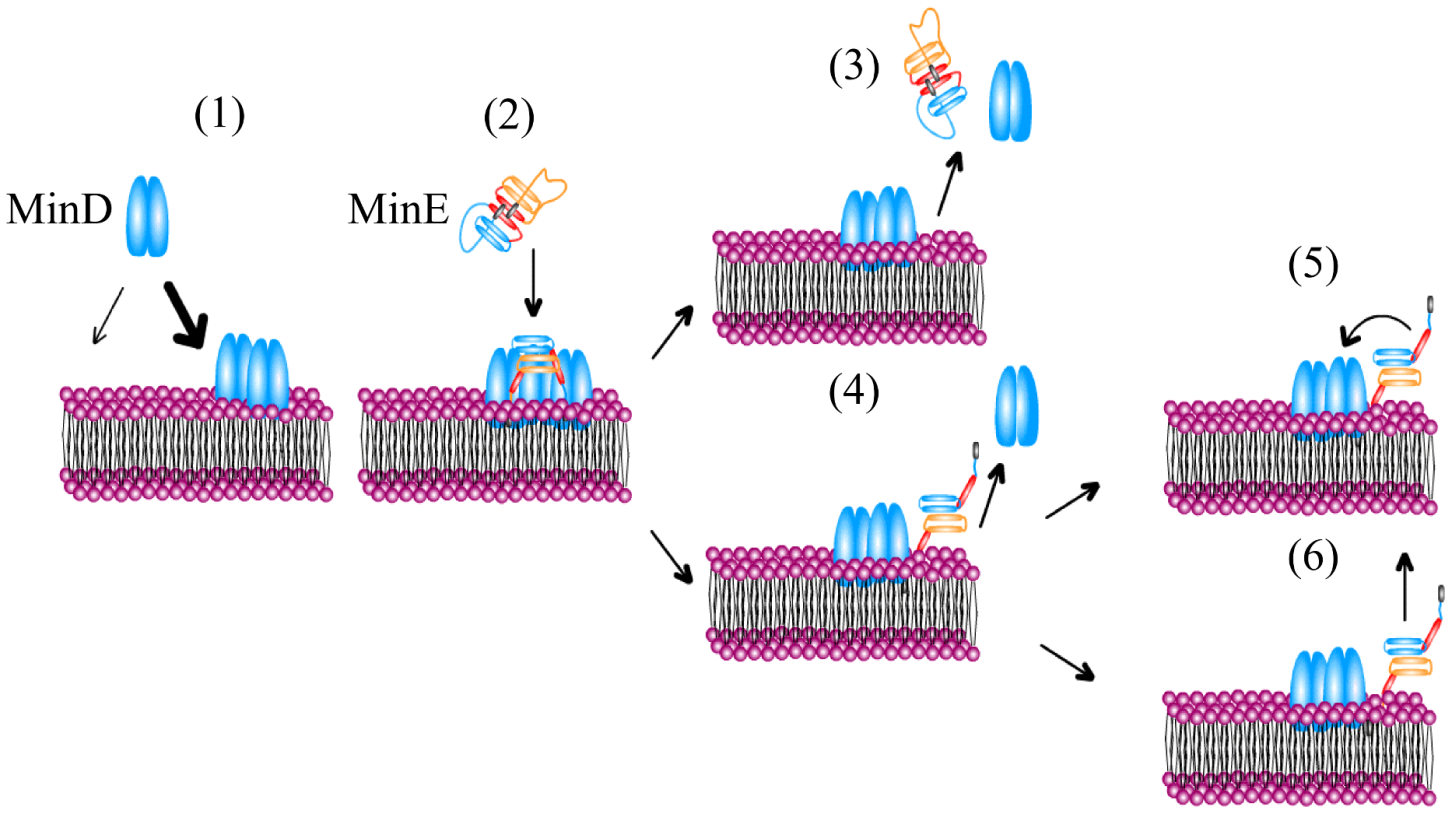
Schematic illustration of the molecular processes involving MinD, MinE, and the membrane. Cytosolic MinD dimers bind to the membrane, with an increased rate in the vicinity of membrane-bound MinD (1). Note, that the molecular mechanism underlying cooperative membrane binding of MinD has not been characterized yet and it is still unclear whether or not membrane-bound MinD form clusters. Cytosolic MinE bind to membrane-bound MinD and form MinDE complexes (2). MinDE complexes dissociate in one of two different ways: MinD and MinE detach simultaneously from the membrane (3) or MinD detaches whereas MinE remains on the membrane (4). There it can rebind to another MinD protein (5) or detach (6).

The processes captured in our analysis were the following: MinD in the vicinity of the membrane associates at a rate 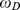 with the lipid bilayer, see Figure 2. Cooperative effects in the binding process lead to an increase of the binding rate if membrane-bound MinD are present nearby. We capture this effect through increasing the binding rate by 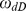 times the local density of membrane-bound MinD.

MinE binds to membrane-bound MinD and forms a MinDE complex [43]. This process occurs at a rate 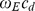, where 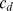 is the local density of membrane-bound MinD. A MinDE complex can dissociate in two ways: either, both, MinD and MinE, detach from the membrane or only MinD leaves the membrane, whereas MinE stays on the lipid bilayer. The two processes occur at rates 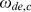 and 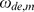, respectively. Individual MinE dimers on the membrane associate with nearby membrane-bound MinD at rate 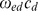 or dissociate from the membrane at rate 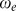.

Finally, all molecules can diffuse in the cytoplasm or on the membrane. Let us emphasize, that we ignore any spatial heterogeneities due to variations in the lipid composition of the membrane, to cytoplasmic crowding in the region of the nucleoid, or to the possible formation of MinD clusters on the membrane. We expect these effects to be of minor importance compared to the processes we consider [44].

To study the patterns resulting from these processes theoretically, we employed two different approaches. On one hand we used a meanfield approach that leads to a system of partial differential equations. On the other hand we used a particle based stochastic model. In this model, each dimer is represented by a particle that moves randomly in space and the processes mentioned above occur stochastically. The corresponding reaction schemes are
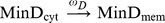
(1)

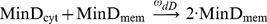
(2)

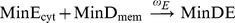
(3)

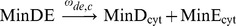
(4)

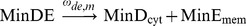
(5)

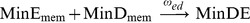
(6)

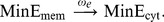
(7)Furthermore, we include the fact that the density of membrane-bound MinD is limited such that the rate of MinD attachment to some membrane area is proportional to the number of free binding sites in that area.

In the meanfield approach the state of the system is given by densities for the various protein states. The volume densities 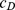 and 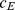 denote the cytosolic concentrations of MinD dimers and MinE dimers, respectively. The surface densities of membrane-bound MinD, MinE, and MinDE complexes are denoted by 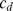, 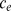, and 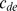, respectively. The time evolution of these densities is governed by the following dynamic equations
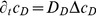
(8)

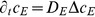
(9)

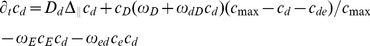
(10)

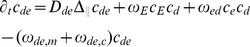
(11)

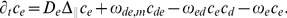
(12)The densities 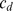, 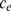, and 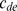 are defined only on the surfaces representing the membrane. In Equations (10)–(12), 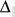 denotes the Laplace-operator on the surface and 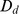, 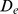, and 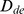 are the respective diffusion constants of membrane-bound MinD, MinE, and MinDE. Furthermore, 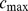 is the maximal MinD density on the membrane. In Equations (10) and (11), the densities 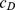 and 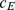 are evaluated at the same points as the surface densities. In Equations (8) and (9), Δ denotes the Laplace-operator in three dimensions and 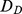 and 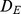 are the diffusion constants for cytosolic MinD and MinE, respectively. The dynamic equations for cytosolic MinD and MinE are complemented by boundary conditions on the diffusion currents that account for protein binding to and detachment from the membrane: The components of these currents orthogonal to the membrane equal the net attachment rate. Formally, we have
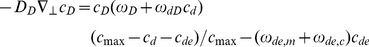
(13)

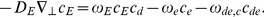
(14)Here, 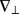 denotes the outward gradient normal to the boundary. Note, that these equations conserve the total protein number.

### Min-protein patterns in cellular geometries

We first studied the behavior of Min protein patterns in cellular geometries. To this end, we solved the stochastic and deterministic dynamic equations in a cylindrical domain with hemispherical caps. The parameters used in this section are given in Table 1. The values of the cytosolic diffusion constants have been measured in Ref. [45]. While there is no direct measurement of the diffusion constants for membrane-bound MinD, MinE, and MinDE, diffusion on membranes is usually two to three orders of magnitude smaller than in the bulk [46]. For larger values of these constants, the resulting patterns are broader and less well defined. Decreasing their values does not affect the patterns significantly. To determine the value of the maximal density of membrane-bound proteins, we use that close packing of MinD on the membrane would yield a density of about 1/(lateral extension of a MinD dimer), with the latter being approximately 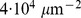. To account for crowding of the membrane by other molecules we use a value roughly 10 times smaller, 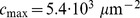. The values of the various attachment and detachment rates have been chosen to match the experimentally observed patterns. Note, that for the parameter values given in Table 1, the dominant path for MinE-induced MinD detachment involves MinE staying on the membrane. This corresponds to a high MinE processivity. Finally, we mostly considered the Min patterns in geometries of fixed size. Even under optimal growth conditions, *E. coli* gains only about 100 nm per oscillation period. As we show below, the patterns are robust against such changes.

**Table 1:** Parameter values used for the numerical solutions of the deterministic dynamic equations (8)–(14) and for the simulations of the stochastic dynamics (1)–(7).

	*in vivo*	*in vitro*	growing cell (1d)
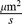	14 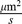	50 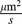	14 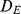
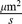	14 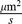	50 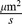	14 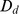
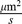	0.06 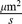	0.3 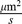	0.06 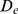
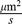	0.3 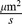	1.8 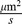	0.3 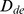
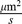	0.06 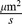	0.3 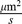	0.06 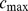
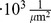	5.4 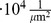	2.75 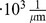	5.4 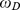
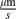	0.1 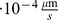	5 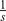	0.1 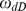
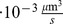	8.8 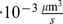	3.18 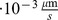	8.8 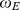
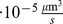	6.96 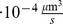	1.36 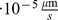	6.96 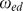
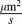	0.139 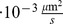	4.9 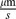	0.139 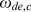
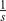	0.08 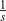	0.16 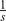	0.08 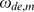
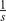	1.5 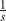	2.52 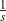	1.5 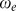
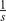	0.5 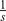	0.5 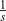	0.5 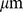

For the cellular geometries, the ‘cell’ diameter was 0.8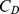. The total MinD and MinE concentrations, 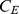 and 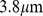, and the system length varied between simulations and are given in the corresponding figure captions.

#### Pole-to-pole oscillations - Standing waves

**Figure 3:**
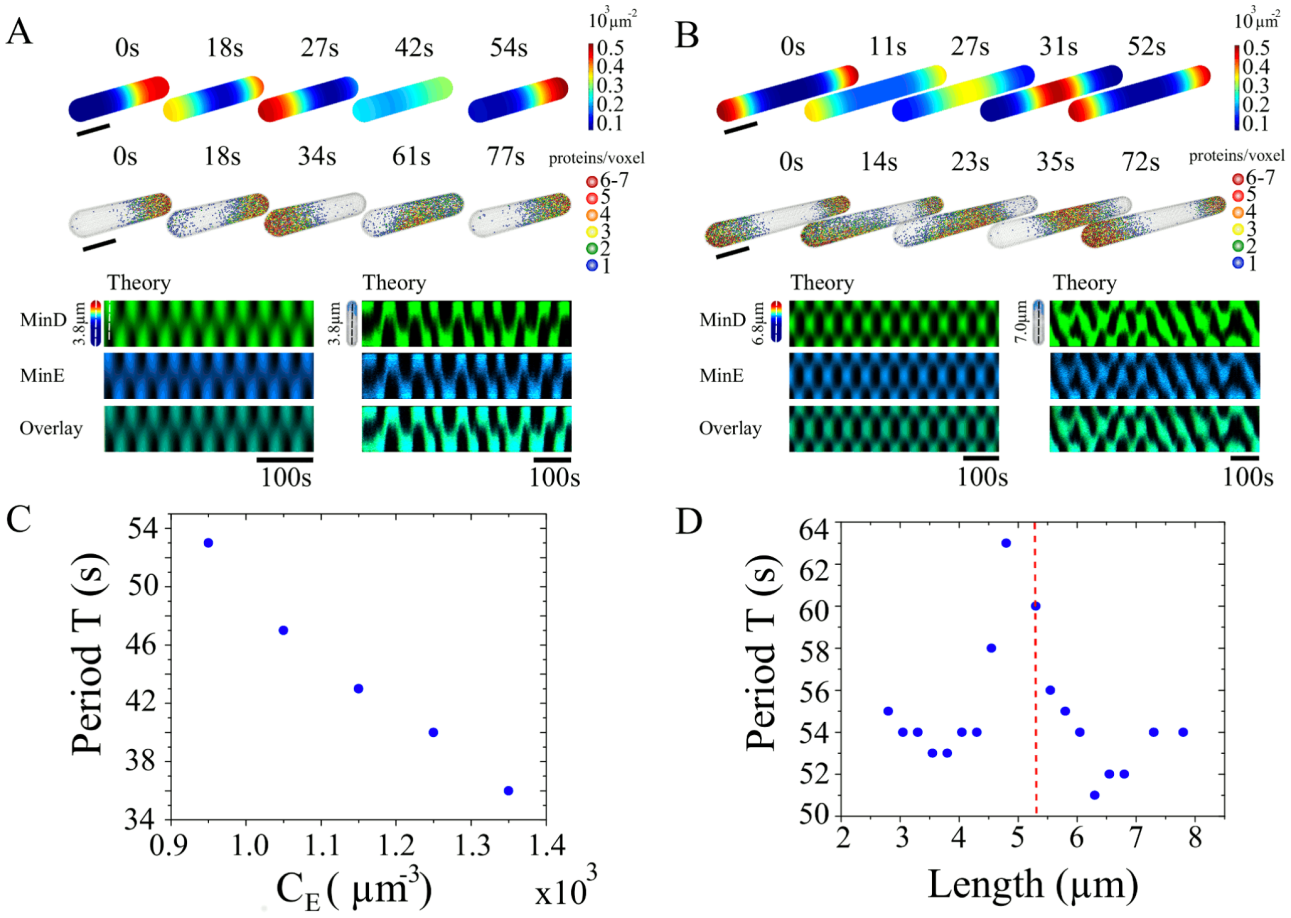
Standing wave patterns from simulations in a bacterial geometry. A) Pole-to-pole oscillations for a system of length 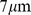. B) Standing wave with two nodes for a system of length 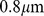. The diameter is 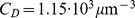 in both cases. Top rows: Distributions of membrane-bound MinD for the deterministic system; middle: same for the stochastic system; bottom: corresponding kymographs. C) Deterministic oscillation period as function of the total MinE concentration with 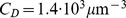. D) Deterministic period as function of the system length with 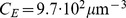 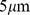. The dashed vertical line indicates the length at which the pattern changed from pole-to-pole oscillations to a standing wave with two nodes.

The pole-to-pole oscillations described in the Introduction are physiologically the most important patterns formed by the Min proteins. In Figure 3A and Movie S1, we show that for total protein concentrations similar to those in wild type *E. coli* and for a cell length of 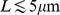, our dynamic equations reproduce this pattern. The oscillation period is about 50s, comparable to experimental values. The pattern does not change qualitatively as long as the system length *L* obeys 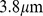. In agreement with previous work [47], [48], stochastic simulations of the processes described in Eqs. (1)–(7) show, that molecular noise does not destroy this pattern.

If the cell length is increased beyond 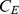, then the pattern changes. In this case, the Min proteins still form a standing wave, but the number of nodes is larger than one, see Figure 3B and Movies S2, S3. This result agrees with the experimentally observed Min-protein patterns in long cells. The appearance of multiple nodes has its origin in the characteristic length scale of the Min-protein patterns that is also evident from the *in vitro* patterns reported in Ref. [10], which we discuss below.

In Figure 3C and D, we present the oscillation period as a function of the total MinE concentration 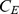 and of the system length, respectively. It decreases approximately linearly with increasing 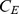, reflecting the increasing activity of MinE removing MinD from the membrane. The dependence on cell length is non-monotonic. Overall, the dependence of the period on the system length is less pronounced than its dependence on 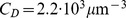. Combining the data from Figure 3C, D we conclude that the oscillation period is not a robust feature of the Min system. This conclusion is in line with experimental measurements of the oscillation period as a function of cell length *in vivo*, which showed significant differences between different cells [4], [19].

#### Traveling waves

**Figure 4:**
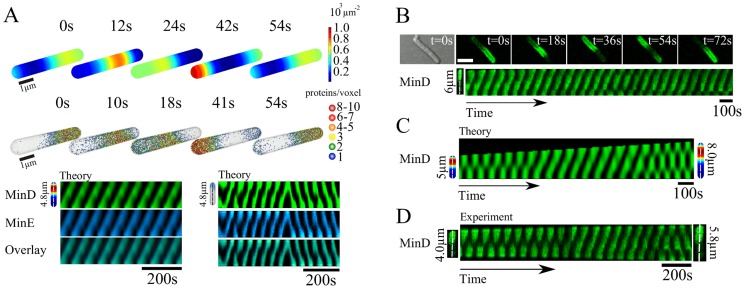
Traveling Min-protein waves in bacterial geometries. A) Traveling wave solutions to the deterministic (top) and stochastic (middle) dynamic system for total protein concentrations of 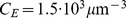 and 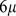. Bottom: corresponding kymographs. B) Distribution of MinD-GFP in a living cell of length 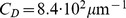
m. C) Kymograph of the MinD distribution in a simulated growing one-dimensional cell. The total protein concentrations are 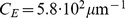 and 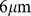. D) Distribution of MinD-GFP in a growing cell.

Changes in the self-organized Min-protein pattern can also be induced by changing the total MinD and/or MinE concentrations. As shown in Figure 4 and Movie S4, for a total concentrations of 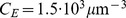 and 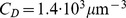 compared to 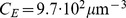 and 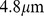 used above, we find traveling waves in cells of 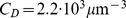 length. In these states, the Min proteins assemble at one cell pole and then travel along the membrane towards the opposite pole. There, the proteins detach from the membrane and move through the cytoplasm back towards the original pole where they assemble again on the membrane and restart the process. In longer systems, the traveling wave breaks up into packets moving in the same direction reflecting the wave length inherent to the dynamic system. As expected on the ground of the system's symmetries, we occasionally observed in the stochastic simulations a change in the direction of motion of the traveling waves, see Figure 4A.

Earlier anecdotal reports of traveling Min protein waves have been given by Shih et al. [49], who mentioned the occasional drift of a Min-protein band from one pole to the other for minE^D45A/V49A^ *E. coli*, and by Tostevin and Howard [50], who observed traveling bands in irregular patterns generated by stochastic simulations. We used fluorescence microscopy to examine the MinD distribution in cells expressing MinD-GFP, see Materials and Methods. In cells with lengths above 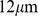 we could indeed observe traveling waves as predicted by the dynamic equations, see Figure 4B. Furthermore, in cells of about 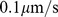 length we observed two wave packets, see Movies S5, S6. We can compare the traveling waves observed *in vivo* with those found *in vitro*. The experimentally measured wave velocity *in vivo* is about 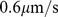 compared to roughly 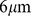 *in vitro*, whereas the wave length *in vivo* is about 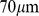 *in vivo* and 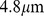 *in vitro* [10], [22]. The ratios of the wave velocities and lengths are thus comparable.

Our calculations pointed to another situation, where traveling waves should be observable. In systems growing in length, traveling waves appeared typically around the critical length where a standing wave with *n* nodes turned into one with *n*+1 nodes, see Figure 4C. Also this prediction is confirmed by experiments: in long recordings of the Min distribution in living *E. coli*, where we could observe a change between different standing wave patterns, we observed transiently traveling waves,see Figure 4D. For the calculations we solved the dynamic equations in one spatial dimension. The corresponding dynamic equations are presented in the Text S1.

#### Phase diagram

**Figure 5:**
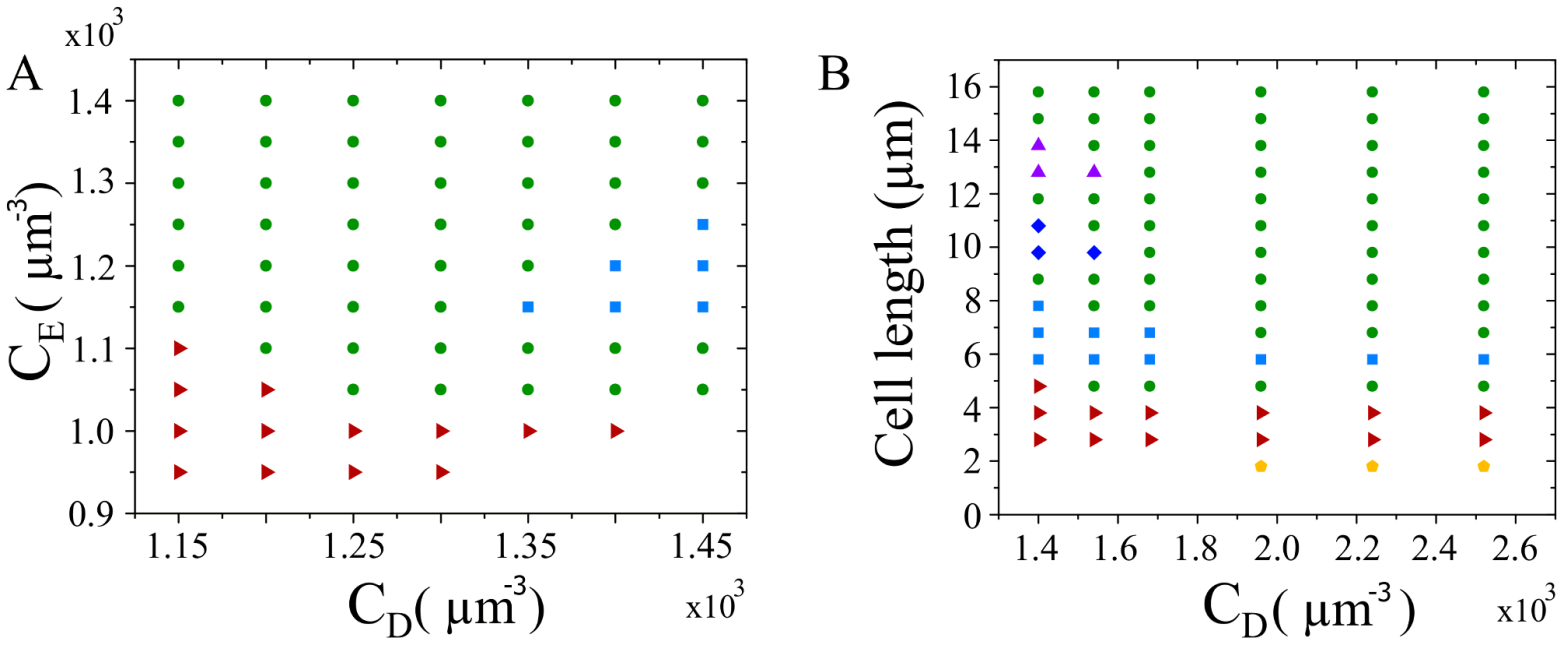
Phase diagram. Min protein patterns in cellular geometry with length 4.8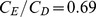 for varying total MinD and MinE concentrations (A) and for varying total MinD concentration and length with 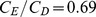 (B). Symbols represent pole-to-pole oscillations (red triangles), traveling waves (green circles), standing waves with two nodes (light blue squares), spatially heterogeneous steady states (yellow pentagons), and standing waves with three (dark blue diamonds) and four nodes (purple triangles). Parameters see Table 1.

To obtain a comprehensive picture of the various states the Min system can generate, we present in Figure 5 cuts through the system's phase diagram obtained from numerical solutions of the dynamic equations (8)–(12). Let us first discuss the influence of the total MinD and MinE concentrations on the pattern in a cell of fixed length of 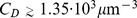, see Figure 5A. For total concentrations of MinE below a critical value, the distributions were homogenous. For higher concentrations, standing waves emerged. They turned into traveling waves for even higher MinE concentrations. For 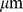, standing waves with two nodes emerge in a finite interval of total MinE concentrations.

**Figure 6:**
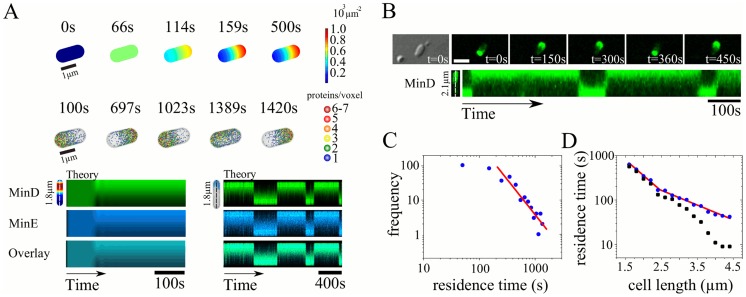
Stationary patterns and stochastic switching. A) Pattern for 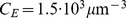 and 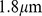 in a system of length 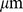 obtained from the deterministic (top) and the stochastic system (middle). Bottom: corresponding kymographs. B) Distribution of MinD-GFP in a living cell of length 2.1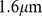. C) Distribution of residence times in the stochastic switching regime from simulations of 12000s in systems of 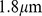, 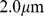, 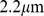, 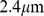 and 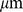 length. D) Average residence time (blue dots) and standard deviation of the corresponding distributions (open squares) obtained from simulations as a function of cell length. Lines represent exponential functions with characteristic lengths of 1.45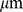 and 3.0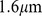, respectively.

In Figure 5B, we present the phase diagram as a function of the total MinD concentration and of the system length, but for fixed ratio of the total MinD/MinE concentrations, 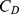. For sufficiently low values of 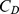 standing waves with an increasing number of nodes appear as the system length is increased. Standing wave patterns with different numbers of nodes are separated by traveling waves. With increasing values of 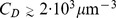, standing waves with several nodes cease to exist. Instead a new state appears in sufficiently short systems for 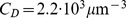. There the distributions are stationary but not homogenous. In that case, the system spontaneously breaks the mirror symmetry with respect to the cell center. They correspond to situations in which most proteins reside in one cell half and two mirror solutions coexist, see Figure 6A.

#### Stochastic switching

In the examples discussed so far, the effects of molecular noise on the Min patterns were minor. This is in agreement with previous work [47], [48]. There are some situations, however, in which noise is essential to understand the emerging Min-protein pattern. In cells lacking the negatively charged lipid phosphatidylethanolamine (PE), pole-to-pole oscillations are suppressed [33]. Instead small spots of membrane-bound MinD form stochastically on the cytoplasmic membrane. Furthermore, our analysis of the mean-field equations (8)–(12) had shown the existence of mirror-symmetric stationary states in short cells. In a stochastic system one might expect that the proteins switch stochastically between these two states. Indeed, there is a critical cell length below which the Min proteins do not oscillate, but switch stochastically between the two cell poles in case MinD and MinE are overexpressed [7], [8], see Figure 6B and Movie S7.

As in experiments, the switching time is very short compared to the time the proteins spend in one cell half. In Figure 6C, we present the distribution of the corresponding residence times. The distribution decays algebraically with a slope of −2.06±0.27. This value is very similar to the experimental value of −2.1. In Figure 6D, we show the dependence of the mean residence time on cell length. Two regimes can be distinguished. For system lengths between 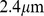 and 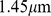 the mean residence time decays exponentially with a characteristic length of 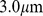. It then turns sharply into an exponential dependence with a characteristic length of 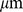. Before the transition, the standard deviations of the distributions of residence times are comparable to the respective mean values. After the transition the standard deviation decreases more rapidly than the average residence time, indicating an increasing regularity of the pole-to-pole oscillations. This is qualitatively similar to observations *in vivo* [7]. The characteristic lengths agree within a factor of three with the experimental values.

#### Min patterns in aberrantly thick cells

**Figure 7:**
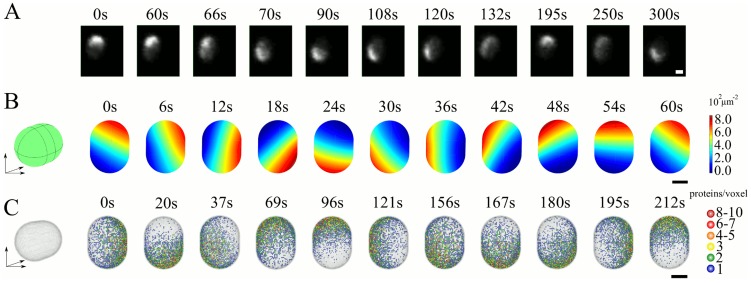
Min patterns in aberrantly thick cells. A) Distribution of MinD-GFP in living cells after exposition to A22 (snapshots from time-lapse fluorescence imaging). B) Solution to the deterministic dynamic equations. C) Solution to the stochastic dynamic equations. In (B) and (C), the system has a diameter of 2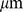 and a length of 2.7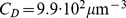, the total MinD and MinE concentrations are 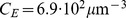 and 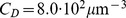 in (B) and 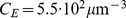 and 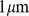 in (C). Other parameters as in Table 1. Scale bars: 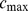.

All patterns in the bacterial geometry discussed so far were invariant under rotations with respect to the systems long axis. One might expect that the Min-protein patterns will break this symmetry if the cell diameter is sufficiently large. It is possible to increase the cell diameter by destroying the MreB filaments that regulate the growth of the cell wall through the application of A22 to living *E. coli* [51]. After treatment with A22, we observe a localized accumulation of MinD moving on the cytoplasmic membrane, see Figure 7A. Note, that the direction of motion of the spot changes in this process, see Movie S8. In former works, the effects of cell size on the Min-protein patterns have been studied in round *rodA* mutants [52] and Δ*mreB* cells [53]. In the first case, irregular oscillations were observed, whereas in the second case mostly regular oscillations, but also MinD spots moving around the cell circumference were reported.

Solving the dynamic equations (8)–(12) in a geometry corresponding to cells after A22 treatment, we also observe that the patterns' rotational symmetry with respect to the system's long axis is spontaneously broken, see Figure 7B, C and Movie S9. In that case, a spot forms at one cell pole. It then travels at constant speed along a planar path through the two cell poles. These patterns are distinct from helical waves generated in thick cells reported for an aggregation-current model of the Min protein dynamics [54]. The behavior is similar to the one observed experimentally. In the deterministic calculations, though, the spot moves along a well-defined closed path without changing its direction of motion. This is different for the stochastic solution, see Figure 7C, where the spot frequently changes direction after passing a cell pole.

### Min protein patterns in open geometries

A major breakthrough in the understanding of Min-protein pattern formation has been achieved by studying the Min-dynamics in open geometries [10], [22], [26], [54]. Experimentally, *in vitro* studies using supported lipid bilayers have allowed us to clearly establish the propensity of the Min proteins to self-organize [10]. Structural analysis suggested that binding to the membrane can also occur for MinE not associated with MinD [23], providing a natural explanation for guiding Min-protein waves on structured surfaces [26].

**Figure 8:**
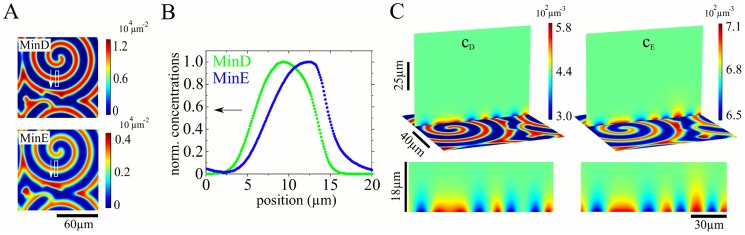
Solutions to the deterministic dynamic equations in the *in vitro* geometry. A) Membrane-bound MinD- and MinE-densities on a planar surface with periodic boundary conditions. B) Density profiles of MinD and MinE obtained from the white rectangle indicated in (A). C) z-dependence of the cytosolic MinD and MinE densities. Top: Buffer concentrations along a slice in the system, bottom: close-up of the buffer concentrations in this slice. Periodic boundary conditions were applied along the *x*- and *y*-axes, no flux boundary conditions on the diffusion current in *z*-direction at 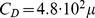. The total MinD and MinE concentrations are 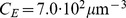
m^−3^ and 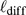. Parameter values are given in Table 1.

In Figure 8 we present the result of a numeric solution of the dynamic equations (8)–(12), where we have employed periodic boundary conditions in the *x*- and *y*-directions. Parameter values are given in Table 1. The differences between these values and those used for the *in vivo* geometries reflect differences in the environmental conditions, notably the presence respectively absence of other macromolecules. Similar to the experimental observations, the Min proteins self-organize into traveling waves. The calculated wave profile presents the same features as in the experiment: the MinD profile increases at the wave front and then saturates until it sharply drops. The density of MinE increases more slowly than that of MinD. Towards the wave's trailing edge it exhibits a sharp increase and then drops rapidly. The parameter 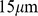 is increased in comparison to the value determined in the section ‘Min-protein patterns in cellular geometries’. The presentation of the distribution's z-dependence in Figure 8C shows that the pattern is confined to a layer of about 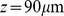 above the membrane. This result justifies a posteriori the use of effective 2d descriptions for the Min-protein dynamics [10], [26] even though it is not obvious how to formally obtain the 2d equations from the 3d system.

### Intuitive picture of Min-protein patterns

The propagation of the wave fronts can be understood by interpreting the space coordinate in Figure 8B as time: First cytosolic MinD binds to the empty membrane. The nonlinearity in the MinD binding term then leads to an increased binding rate and thus to an accelerated increase of the MinD density on the membrane. As soon as membrane-bound MinD is present, MinE starts to attach. As the MinE binding sites are abundant, the increase of the MinE density is roughly linear. With increasing MinE density, the net rate of MinD attachment decreases. Eventually, the MinE-induced detachment rate exceeds the attachment rate and the density of membrane-bound MinD decreases. This decrease is sharp at the waves trailing edge, because MinE processivity leads to an accumulation of MinE in this region.

**Figure 9:**
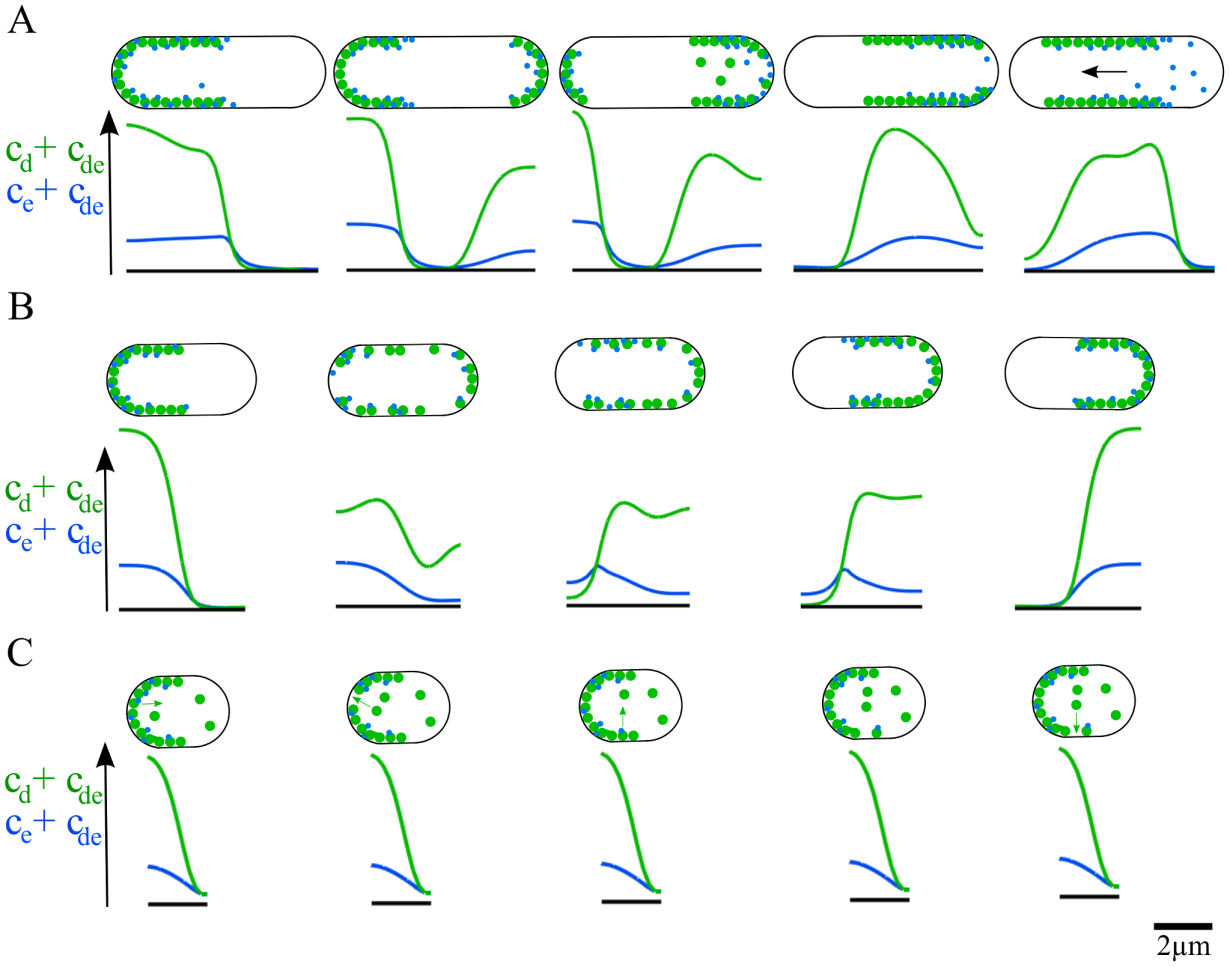
Snapshots of MinD and MinE distributions in a one-dimensional system and corresponding illustrations. A) If the diffusion length of MinD is of the same order as the cell size, the MinD density will increase towards the right pole. The same holds for MinE and a wave traveling towards the left is initiated. B) If the diffusion length is larger than the cell, a second maximum in the MinD distribution close to the left pole will form. It adsorbs most of the free MinE and a wave will start from the center towards the left pole. C) For MinD diffusion lengths that are very large compared to the cell size MinD rebinds only in the left cell half and a stationary state results. Parameters as in Figure 4C.

The sequence of Min protein patterns *in vivo* upon changing the system length can be intuitively understood from the mechanism underlying traveling waves *in vitro*. To this end, we introduce the diffusion length 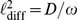, which is the length a molecule typically diffuses before attaching to the membrane. For a diffusion constant *D* and an attachment rate *ω* it is given by 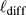. Now, consider a wave in a cell propagating in the direction of the long axis. The wave is sustained by molecules binding to the wave's leading edge after they have been released from the trailing edge. When the wave reaches a pole, the MinD dimers released from the membrane at the trailing edge can no longer bind at its leading edge. Instead, they diffuse away form the cell pole. If the cell length is on the order of 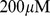, the proteins will preferentially bind at the opposite pole [55], see Figure 9A. Similarly, with some delay, MinE released from the original wave, will bind at this pole, too, and a new wave traveling in the same direction as the original one is generated.

If the system size is shorter, MinD binding will occur in a zone extending further from the new pole to the cell center because the ratio of diffusion length to the cell length has increased. As the affinity for MinE binding to MinD on the membrane is large, MinE will preferentially bind to the part of the MinD zone proximal to the cell center and the wave will move into the opposite direction compared to the original wave, see Figure 9B, thus giving rise to pole-to-pole oscillations. For even shorter cells, the distribution of cytosolic MinD and MinE is essentially homogenous as the diffusion lengths significantly exceeds the cell length. MinD and also MinE thus bind preferentially to zones of the highest MinD concentrations on the membrane and a stationary profile emerges, see Figure 9C.

The picture presented here is thus somewhat different from the mechanism underlying the pole-to-pole oscillations proposed in Ref. [15] as we discuss below. Let us finally note, that it is harder to get an intuitive picture of the dependence of the Min-protein patterns on the total protein concentration and we refrain here from discussing this topic further.

## Discussion

In this work, we presented a computational study of self-organized pattern formation by MinD and MinE from *E. coli*. The equations, which notably account for membrane-binding of MinE, generate the patterns previously observed in living cells as well as Min protein waves on flat surfaces observed in reconstitution experiments. In addition, our analysis yielded two patterns that had not been reported before: In sufficiently long cells and for elevated protein levels, traveling waves emanating from one cell pole and propagating to the opposite pole should emerge. Secondly, in aberrantly large cells, the rotational symmetry of the pattern should be lost and a moving spot should form instead. Both predictions were confirmed experimentally. We conclude that the membrane-binding of MinE is an essential molecular feature to comprehensively describe large-scale pattern formation of the Min proteins.

*In vitro* experiments on micropatterned membranes suggest an important role of MinE processivity for Min-protein pattern formation [26], but it remains to be seen whether this is the case *in vivo*. In fact, comparing our system to the one proposed by Huang et al. [15] shows that MinE processivity can at least in part be replaced by a high rate of MinE binding to membrane-bound MinD (they chose a rate orders of magnitude higher than we did). This leads to a different mechanism underlying the pole-to-pole oscillations and requires a finite MinD-ADP to MinD-ATP exchange rate for stabilizing standing waves with several nodes. It will be interesting to test experimentally which of the two possibilities is realized in living *E. coli*.

Our description neglects many molecular details. For example, we did not consider explicitly a MinD dimerization step or the finite exchange rate of ADP for ATP for cytosolic MinD. Also, different expressions accounting for the binding of cytosolic MinE to membrane-bound MinD might be used. We analyzed several different expressions describing the effect that a single MinE dimer can induce detachment of several MinD dimers from the membrane. While these modifications led to quantitative differences, their analysis also revealed that details of the corresponding expressions are rather unimportant for the overall behavior of the system.

As a consequence of the relatively simple reaction terms employed in our description, our model reveals some quantitative discrepancies compared to experimental observations. For example, the fluctuations present in the kymographs in Figure 3A and B are apparently larger than in the experimental kymographs in Figure 1. In addition, the wave profile shown in Figure 8 differs from the experimentally determined [22]. However, complete quantitative agreement likely requires knowledge of more molecular details of the reactions involved. Note, however, that a quantitative comparison on the single cell level also requires precise measurements of the corresponding amount of MinD and MinE, which are currently not available. On a coarser level, though, our description seems to match the topology of the phase space. That is, we present one set of parameters, that correctly reproduces the sequence of patterns as cells grow and also correctly describes the appearance of stochastic switching and traveling waves in living cells with increasing protein levels. In contrast, the exact transition points differ in general from those observed in experiment and any coincidence would be fortuitous. Let us also emphasize that, experiments are now very much needed to constrain possible parameter values. Only with such data we can expect to make further significant progress in understanding Min protein patterns.

In agreement with previous work, our analysis also showed that molecular noise has only a minor effect on the Min-protein patterns. Macroscopic signatures of molecular noise were only found under special conditions, namely, in short cells presenting stochastic switching and in large cells, where the Min proteins formed a rotating patch with a stochastically switching sense of rotation. Our description of the Min-protein dynamics can now be used to to design new experiments, for example, to test the interplay between the Min oscillations and Z-ring assembly *in vivo* or to determine conditions to generate Min-protein patterns inside vesicles *in vitro*. Such experiments could present important steps on the way to synthesize a system that is able to divide autonomously, that is, a minimal synthetic cell.

## Materials and Methods

### Experiments

We used cells of the *E. coli* strain JS964 containing the plasmid pAM238 encoding for MinE and GFP-MinD under the control of the lac-Promoter [5]. Bacteria were grown overnight in a 3ml LB medium at 37°C. Cells were induced with Isopropyl – β – D-thiogalactopyranosid (IPTG) at a concentration of 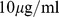 and incubated for 3–4 hours prior to measurements. During 1–2 hours prior to measurement, cells were kept at 30°C for better fluorescence. The optical density was less than 0.6. During measurements, cells were in the exponential growth phase. The samples were kept at a temperature of 30°C using a Bachhoffer chamber. To keep bacteria from moving under the cover slip, we put them on an agar pad (1% agar solution in LB medium with a reduced yeast extract fraction, 10%, in order to lower background fluorescence). The fluorescence recordings were taken with an Olympus FV 1000 confocal microscope, at an excitation wavelength of 488 nm from a helium laser at low power. We used an Olympus UPLSAPO 60×, NA 1.35 oil immersion objective and recorded a frame every 3s. A measurement lasted 40min. During this period, the focus was manually readjusted at irregular intervals. A22 (S-(3,4-Dichlorobenzyl)isothiourea, HCl) was purchased from Merck Millipore. Cells were imaged 2–3 hours after adding 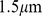 of A22.

### Numerical solutions of the dynamic equations

We solve the dynamic equations (8)–(14) in the *in vitro* as well as in the *in vivo* geometry by using Comsol Multiphysics 4.1 which is a solver for partial differential equations based on the finite element method (FEM). All computations with exception of those for Figure 4C were performed in 3d and no assumption was made about the symmetries of the solutions. For the calculations for the patterns in a bacterial geometry the maximal grid size was 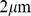. For the calculations in the *in vitro* geometry, we used a maximal grid size of 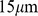 in the surface domain and of 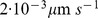 in the buffer domain. As initial condition we used homogenous distributions of cytosolic proteins with a random perturbation of 5–10%. The initial surface densities were chosen to be zero for the *in vivo* geometries. For the *in vitro* geometry the surface densities were different from zero in a semi-annulus to rapidly induce a spiral.

The calculations for the growing cell presented in Figure 4C, the system length was increased by adding discrete pieces at one end of the interval. For the pattern shown in Figure 4, the rate of growth was 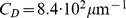. On the added pieces, the protein densities of cytosolic MinD and MinE were initialized with the values 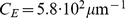 and 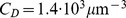, respectively, whereas the densities of membrane-bound proteins were initially set to zero.

To simulate the stochastic reaction diffusion kinetics (1)–(7) in three dimensions, we used MesoRD [56], a tool to solve the stochastic Master Equation using a reaction diffusion Master Equation. It is based on the Next Subvolume Method [57].

## Supporting Information

Text S1 Lower dimensional versions of the dynamic equations and Min-protein pattern formation in growing cells.
(PDF)

Video S1(Theory) Pole-to-pole oscillation for a system of length 3.8 μm. The total protein concentrations are 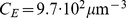 and 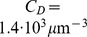.(AVI)

Video S2(Theory) Standing wave with two nodes for a system of length 7 μm. The total protein concentrations are 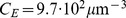 and 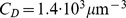.(AVI)

Video S3(Theory) Standing waves with 3 and 4 nodes. The systems have a length of 10 μm and 13 μm. The total protein concentrations are 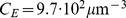 and 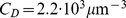.(AVI)

Video S4(Theory) Traveling waves in a cell of 4.8 μm length. The total protein concentrations of 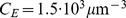 and 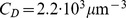.(AVI)

Video S5 (Experiment) Time-lapse fluorescence microscopy of MinD-GFP showing traveling waves in living *E. coli* cells.
(AVI)

Video S6(Theory) Traveling wave with two wave packets. The length of the cell is 12 µm and the total protein concentrations are 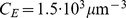 and 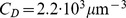.(AVI)

Video S7(Theory) Stationary pattern and stochastic switching. The length of the system is 1.8 μm and the total protein concentrations are 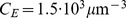 and 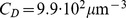.(AVI)

Video S8 (Experiement) Time-lapse fluorescence microscopy of MinD-GFP showing traveling waves in a living *E. coli* cell after treatmemt with A22, see Materials and Methods.
(AVI)

Video S9(Theory) Min-protein pattern formation in a aberrantly large cell. The system has a length of 2.7 μm and a diameter of 2 μm. In the deterministic simulation, the total protein concentrations are 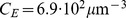 and 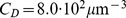 and in the stochastic simulations 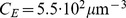 and 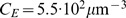.(AVI)

Video S10 (Theory) Simulation of Min-protein pattern formation in the 3D *in vitro* geometry. (A) z-dependence of the cytosolic MinD and MinE densities. (B) Densities of membrane-bound MinD and MinE on a planar membrane with periodic boundary conditions.
(AVI)
