# Supplementary material for: Membrane Binding of MinE Allows for a Comprehensive Description of Min-Protein Pattern Formation
Source: PLoS Comput Biol. 2013 Dec 5;9(12):e1003347. doi: 10.1371/journal.pcbi.1003347 (PMC3854456; doi:10.1371/journal.pcbi.1003347)
Supplement: Text S1 — Lower dimensional versions of the dynamic equations and Min-protein pattern formation in growing cells. (PDF) [file pcbi.1003347.s001.pdf]

The role of transient membrane binding of MinE for Min-protein pattern formation

Supporting Information

M. Bonny,[1] E. Fischer-Friedrich,[2,3] M. Loose,[4] P. Schwille,[5] and K. Kruse[1]

[1] *Theoretische Physik, Universität des Saarlandes, Postfach 151150, 66041 Saarbrücken, Germany*
[2] *Max-Planck-Institut für Zellbiologie und Genetik, Pfotenhauerstraße 108, 01307 Dresden, Germany*
[3] *Max-Planck-Institut für Physik komplexer Systeme, Nöthnitzerstraße 38, 01187 Dresden, Germany*
[4] *Department of Systems Biology, Harvard Medical School, 200 Longwood Avenue, Boston, MA 02115, USA*
[5] *Max-Planck-Institut für Biochemie, 82152 Martinsried, Germany*

LOWER DIMENSIONAL VERSIONS OF THE DYNAMIC EQUATIONS AND MIN-PROTEIN PATTERN FORMATION IN GROWING E. COLI

Many aspects of the Min protein dynamics in cellular geometries can be studied using a one-dimensional version of the model, where the dimension is along the cell's long axis. Similarly, the patterns in the *in vitro* geometry obtained from the full dynamic equations in three spatial dimensions presented in the text can also be generated by a two-dimensional version. While it is not obvious how to rigorously obtain the dynamic equations in lower dimensions, we can write easily equations in lower dimensions that account for the same processes as the full dynamic equations. For example, in one spatial dimension, we have

$$\begin{aligned}
\partial_t c_D &= D_D \partial_x^2 c_D - c_D(\omega_D + \omega_{dD} c_d)(c_{\max} - c_d - c_{de})/c_{\max} \\
&\quad + (\omega_{de,m} + \omega_{de,c}) c_{de} && (1)\\
\partial_t c_E &= D_E \partial_x^2 c_E - \omega_E c_E c_d + \omega_e c_e + \omega_{de,c} c_{de} && (2)\\
\partial_t c_d &= D_d \partial_x^2 c_d + c_D(\omega_D + \omega_{dD} c_d)(c_{\max} - c_d - c_{de})/c_{\max} \\
&\quad - \omega_E c_E c_d - \omega_{ed} c_e c_d && (3)\\
\partial_t c_{de} &= D_{de} \partial_x^2 c_{de} + \omega_E c_E c_d + \omega_{ed} c_e c_d \\
&\quad - (\omega_{de,m} + \omega_{de,c}) c_{de} && (4)\\
\partial_t c_e &= D_e \partial_x^2 c_e + \omega_{de,m} c_{de} - \omega_{ed} c_e c_d - \omega_e c_e \quad . && (5)
\end{aligned}$$

The line densities c_D and c_E describe the distribution of cytosolic MinD and MinE along the bacterial long axis, while c_d, c_{de}, and c_e are the corresponding line densities for membrane-bound MinD, MinDE complexes, and MinE, respectively. The system is complemented by using no-flux boundary conditions. For the dynamics in two spatial dimensions representing a flat membrane, analogous equations can be written.

To solve for the Min-patterns in growing cells, we used the 1d description. The length of the interval (cell) was increased by adding discrete pieces at randomly chosen places in the interval. For the pattern shown in Fig. 4, the rate of growth was $2 \cdot 10^{-3} \frac{\mu m}{s}$. On the added pieces, the protein densities of cytosolic MinD and MinE were initialized with the values C_D=$8.4 \cdot 10^2 \mu m^{-1}$ and C_E=$5.8 \cdot 10^2 \mu m^{-1}$, respectively, whereas the densities of membrane-bound proteins were initially set to zero.
